# Recycling of Silicon Carbide Sludge on the Preparation and Characterization of Lightweight Foamed Geopolymer Materials

**DOI:** 10.3390/polym13224029

**Published:** 2021-11-21

**Authors:** Kang-Wei Lo, Ya-Wen Lin, Ta-Wui Cheng, Kae-Long Lin, Wei-Ting Lin

**Affiliations:** 1 Institute of Mineral Resources Engineering, National Taipei University of Technology, Taipei City 106, Taiwan; dark83054689@gmail.com (K.-W.L.); urine1001@gmail.com (Y.-W.L.); twcheng@mail.ntut.edu.tw (T.-W.C.); 2 Graduate Institute of Engineering Technology, National Taipei University of Technology, Taipei City 106, Taiwan; 3 Department of Environmental Engineering, National Ilan University, No. 1, Sec. 1, Shennong Rd., I-Lan 260, Taiwan; 4 Department of Civil Engineering, National Ilan University, No. 1, Sec. 1, Shennong Rd., I-Lan 260, Taiwan; wtlin@niu.edu.tw

**Keywords:** foaming geopolymer, foaming kinetic, fire resistance, silicon carbide sludge, recycling

## Abstract

This study used silicon carbide sludge (SCS) to prepare lightweight foaming geopolymer materials (FGPs) by the direct foaming method. Results showed that when the SCS replacement level was 10%, the bulk density of the lightweight FGPs with added foaming agent amounts of 0.5% and 2.0% was 0.59 and 0.49 g/cm^3^, respectively; at a curing time of 28 days, the lightweight FGPs with amounts of added foaming agent of 0.5% and 2.0% had bulk densities that were 0.65 and 0.58 g/cm^3^, respectively. When the SCS replacement level was 10%, and the amount of added foaming agent was 2.0%, the porosity ratio of the lightweight FGP increased from 31.88% to 40.03%. The mechanical strength of the lightweight FGPs with SCS replacement levels of 10% and 20% was 0.88 and 0.31 MPa, respectively. Additionally, when the amount of foaming agent increased to 2.0%, the thermal conductivity of the lightweight FGPs with SCS replacement levels of 10% and 20% were 0.370 and 0.456 W/m⋅K, respectively. When the curing time was 1 day, and the amount of added foaming agent was 0.5%, the reverse-side temperature of the lightweight FGPs with SCS replacement levels of 10% and 20% were 286 and 311 °C, respectively. The k value of the O_2_ reaction decreased from 2.94 × 10^−4^ to 1.76 × 10^−4^ because the reaction system was affected by the presence of SiC sludge, which was caused the reaction to consume O_2_ to form CO_2_. The results have been proposed to explain that the manufactured lightweight FGPs had a low thermal conductivity (0.370–0.456 W/m⋅K). Therefore, recycling of silicon carbide sludge in lightweight foaming geopolymer materials has potential as fire resistance material for the construction industry.

## 1. Introduction

In recent years, the light-emitting diode (LED) industry was widely used in indicators, and display devices of information [1], communication, and consumer electronic products with the economy and industry were flourishing in Taiwan [2]. A large amount of silicon carbide sludge (SCS) was generated during the process of cutting silicon ingot into wafer. Its cutting loss exceeds 50% [3]; according to the research statistics of Lan et al. [4], the global silicon wafer manufacturing industry spent about 400,000 tons of silicon ingots to produce silicon wafers in 2018. The process of cutting into wafers produced about 200,000 tons of SCS. The SCS contained alumina (Al_2_O_3_) and silicon carbide (SiC), which is the main component of natural kaolin. If SCS can be recycled, it is also reducing environmental pollution and in accordance with the environmental protection administration goal of zero waste and zero landfill resources.

In 1972, geopolymer was originally proposed as a term by Davidovits [5] and described semi-crystalline and three-dimensional (3D) aluminosilicate materials. Geopolymers were generated by the reaction of aluminosilicate materials (metakaolin, fly ash, waste glass, etc.) and highly alkaline activators [6,7]. Foaming geopolymers (FGPs) have a wide range of performance and characteristics, which include high mechanical strength [8], long-term durability [9], low thermal conductivity [10], suitable chemical stability, and thermal stability [11]. Because FGPs are environmentally friendly, they are an ideal substitute for ordinary Portland cement-based porous concrete [12]. FGPs have been proposed as inorganic carriers [13], adsorbents and filters [14,15], catalysts [16], and ecological building materials [17].

The three main techniques used to produce lightweight geopolymer foams are reactive emulsion templating, ice-templating, and direct foaming. The latter is perhaps the most widely used approach and involves chemically and/or mechanically incorporating gas into the paste [18]. At the present state of domestic and foreign research, lightweight FGPs are mainly produced by direct foaming using the chemical or mechanical method [19]. Novais et al. [20] have shown that the incorporation of H_2_O_2_ does not alter the geopolymerization rate, which only depends on the concentration of the alkali solution and the liquid/solid ratio. However, the amount of swelling, apparent density, pore size, homogeneity, and consequently, the final properties of the material vary substantially with the H_2_O_2_ content. Lightweight FGPs have mainly been proposed as a new type of insulating material with a better performance combination (low cost, simple processing conditions, nonflammable, etc.) than traditional insulating materials, such as polyurethane (PU), polystyrene (PS), melamine, foam glass, glass wool and pearlite [9]. Thermal insulation materials have low thermal conductivity and acceptable fire performance and have played a vital role in energy-efficient buildings.

The Waste Electrical and Electronic Equipment (WEEE) Directive, implemented by European Community, attempts to reduce the amount of WEEE produced and encourage reuse, recycling, and recovery, thereby providing an incentive to design electrical and electronic equipment in an environmentally efficient manner that considers waste management. The WEEE Directive also aims to improve the environmental performance of businesses manufacturing, supplying, using, recycling, and recovering waste electrical and electronic equipment. Nevertheless, the objective of this work was to investigate the influence of the H_2_O_2_ was used in combination with SCS to produce lightweight FGPs with low thermal conductivity. Comparative evaluations were investigated via morphology analysis and mechanical strength (flexural and compressive strength) analysis of the foaming characteristics, fire performance, and the macroscopic/microstructural properties of lightweight FGP.

## 2. Materials and Methods

### 2.1. Materials

The SCS used in this study was collected from the blue light-emitting diode (LED) manufacturing plant in Taiwan. The commercially available kaolin is from the Emperor Chemical Co., Ltd. in Taiwan. The SCS was crushed and put into a ball mill for grinding. The fineness was controlled at 300–400 m^2^/kg for the initial material to prepare the SCS-based geopolymer (SCSGP), and the detailed composition is shown in Table 1. The chemical components of the materials are shown in Table 1. included 51.8% SiO2 and 43% Al_2_O_3_. The SiC sludge included 75.4% silica, 23% silicon carbide and 0.8% alumina. Commercially available kaolin was selected and calcined to produce MK at 650 °C for 3 h. The sodium metasilicate solution (Ms = 3.2) was purchased from First Chemical Manufacture Co., Ltd. Solid particles of NaOH (from Thermo Fisher Scientific, United Kingdom) were added to deionized water, allowed to release heat for 24 h, and used to prepare a 10 M NaOH solution. The alkali activator solution was prepared by mixing the 10 M NaOH solution and the sodium metasilicate solution. A hydrogen peroxide (H_2_O_2_) solution was prepared with a concentration of 35 wt.% (from the Nihon Shiyaku Reagent, Japan), and the H_2_O_2_ solution was used as a foaming agent.

### 2.2. Experimental Procedures

At ambient temperatures, the abovementioned alkali activator solution was added to the powder (MK and SCS) and mixed by mechanically mixing to obtain an SCSGP paste. The MK, SCS, and the alkali activator solution were mixed by a laboratory mixer for 7 min to achieve complete homogenization. Subsequently, the SCSGP paste and H_2_O_2_ solution were mixed for 2 min to prepare lightweight FGP. The SCS replacement levels were 0%, 10%, 20%, 30% and 40% in the SCSGP paste. The contents of the H_2_O_2_ solution (used as the foaming agent) were 0.5%, 1.0%, 1.5%, and 2.0% in the lightweight FGP. After mixing, the samples were immediately cast into plastic molds and cured in two steps: (1) the samples were sealed in plastic wrap to prevent the formation of dry cracks, and a constant temperature of 30 ± 2 °C and constant humidity were applied for 24 h; (2) the abovementioned samples were removed from the plastic mold and then further cured under the same conditions for 56 days.

The Archimedes principle was used to determine the bulk density and porosity of the lightweight FGP were measured according to ASTM C373-88 at different curing times. The bulk density (g/cm^3^) of lightweight FGP samples = Bulk density (g/cm^3^) = {dry mass of specimen}/{(saturated surface dry mass of specimen) − (immersed mass of dry specimen)}. The porosity (%) of lightweight FGP samples = saturated mass of specimen − drying the test specimens to constant mass/{(saturated surface dry mass of specimen) − (immersed mass of dry specimen) × 100%} A universal testing machine was used to test the compressive strength (ASTM C109) of the sample. The reported data were the average value obtained for the three specimens. The flexural strength tests were performed after 1, 7, 14, 28, and 56 days using a Hung Ta HT-2402 testing machine with a three-point bending test method at a 5 mm/min crosshead speed, according to ASTM C348. The fire resistance properties were tested following the procedure outlined in ASTM E119-20. The selected samples were broken, and hydration was stopped with absolute alcohol prior to analyzing the microstructure of the sample. The microstructure of the sample was analyzed using FTIR and scanning electron microscopy (SEM). FTIR spectrums were obtained by scanning 2000 to 400 cm^−1^ wavenumbers using the KBr pellet technique (where 1 mg powdered sample was mixed with 150 mg KBr). SEM images were obtained using a Hitachi S-3500 N at an accelerating voltage of 20.0 kV and magnification of 500×.

## 3. Results and Discussion

### 3.1. Physical Property Analysis of Lightweight Fgps

**Figure 1:**
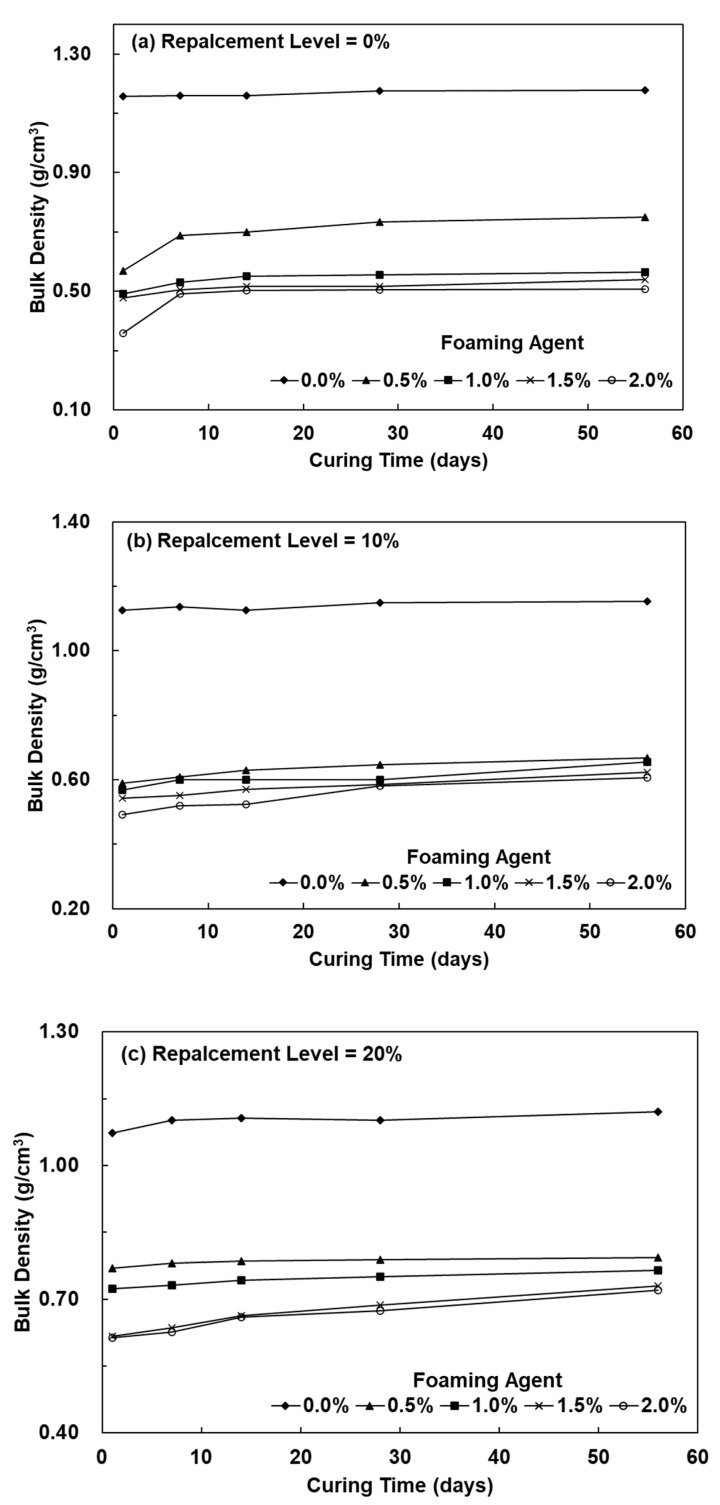
The bulk density of lightweight FGPs with SCS replacement levels. (**a**) 0%; (**b**) 10% and (**c**) 20%.

Figure 1 shows the bulk density of lightweight FGPs prepared with different SCS replacement levels and added H_2_O_2_ solution levels. It can be seen from the figure that when the curing time was 1 day and the SCS replacement level was 0%, the bulk density of the lightweight FGPs with added foaming agent amounts of 0.5%, 1.0%, 1.5%, and 2.0%, was 0.57, 0.49, 0.48, and 0.36 g/cm^3^, respectively. The results showed that the bulk density of the lightweight FGPs decreased with increasing amounts of foaming agents. When the SCS replacement level was 10%, the bulk density of the lightweight FGPs with added foaming agent amounts of 0.5% and 2.0% was 0.59 and 0.49 g/cm^3^, respectively; at a curing time of 28 days, the lightweight FGPs with amounts of added foaming agent of 0.5% and 2.0% had bulk densities that were 0.65 and 0.58 g/cm^3^, respectively. The results showed that the amount of added foaming agent is an important parameter that affects the bulk density of lightweight FGPs. Previously, Ding et al. (2015) used 60 wt.% SiO_2_ aerogel particles to prepare geopolymer insulation materials. The results showed that when the amount of SiO_2_ aerogel particles was 60%, the bulk density was 1.2 g/cm^3^ [21]. The figure shows that for the lightweight FGP with SCS replacement levels of 20%, increasing the amount of foaming agent to 2.0% for 28 days decreased the bulk density from 1.10 to 0.67 g/cm^3^. The bulk density gradually decreased with increasing amounts of foaming agent, but the bulk density still had not significantly changed with increased curing times. It is possible the silicon carbide sludge existed in this environment, the H_2_O_2_ reaction tended to release •OH, and its redox reaction was very strong, accelerating the decomposition of H_2_O_2_ and O_2_ [22,23]; thus, a lightweight FGP with a weak and unstable structure was obtained after foaming, and the results were similar to that of Singh et al. (2020) [21].

**Figure 2:**
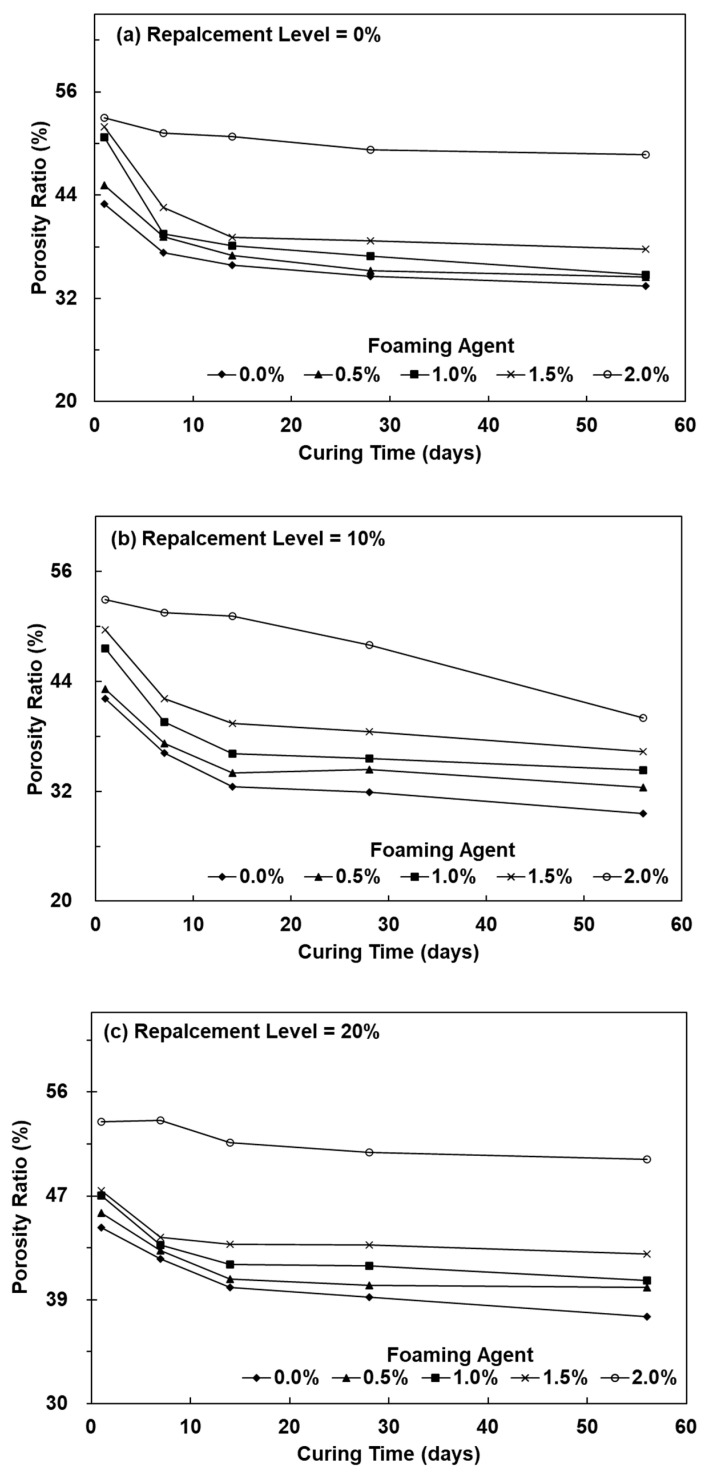
The porosity ratio of lightweight FGPs with SCS replacement levels. (**a**) 0%; (**b**) 10% and (**c**) 20%.

For the lightweight FGP with an SCS replacement level of 0%, increasing the amount of foaming agent to 2.0% for 28 days increased the porosity ratio to 49.26%, as shown in Figure 2. Previous studies have pointed out that for a geopolymer with a high initial concentration of H_2_O_2_, the porosity ratio increased with an increasing volume expansion ratio [18]. When the SCS replacement levels were 10% and the amount of added foaming agent was 2.0%, the porosity ratio of the lightweight FGP increased from 31.88% to 40.03%. The results showed that the porosity ratio increased with increasing amounts of added hydrogen peroxide. Because the system was affected by the presence of SiC, which caused the k value of the O_2_ reaction to decrease, there was a synergistic effect between SiC sludge and metakaolin, which formed more hydration products to fill the pores [24]. In addition, when the SCS replacement levels were 20%, and the amount of added foaming agent was 2.0%, the porosity ratio of the lightweight FGP was 47.96%, which showed that the porosity ratio significantly increased with increasing foaming agent amounts. Novais et al. (2016) used 0.03%, 0.15%, 0.30%, 0.90%, and 1.2% hydrogen peroxide as the foaming agent to produce porous fly ash-based geopolymers. The results showed that the bulk density was 0.6–1.2 g/cm^3^, and the porosity ratio was 42–73% [18]; the porosity ratio results were consistent with our study of a sample with a 2.0% foaming agent.

### 3.2. Mechanical Strength Analysis of Lightweight Fgps

The compressive strength and flexural strength of lightweight FGPs with different SCS replacement levels were studied, and the added H_2_O_2_ solution levels and curing times of 1–56 days are listed in Table 2 and Table 3, respectively. The results show that for the lightweight FGP prepared with an amount of added foaming agent of 2.0% and a curing time of 1 day, the compressive strength and flexural strength were 1.32 and 0.80 MPa, respectively. Because the foaming agent generated bubbles in the system, a weak bearing capacity developed during the mechanical strength test [25], drastically reducing the mechanical strength. When the amount of added foaming agent was 0.5%, the compressive strength of the lightweight FGPs with and SCS replacement levels of 10% and 20% was 1.25 and 0.78 MPa, respectively, which showed a sharp downward trend.

In addition, when the amount of added foaming agent increased to 2.0%, the compressive strength of the lightweight FGPs with SCS replacement levels of 10% and 20% was 0.88 and 0.31 MPa, respectively, and its compressive strength development most slowed. Because the system was affected by the presence of SiC, which caused the k value of the O_2_ reaction to decrease, a synergistic effect existed between the SiC sludge and metakaolin, forming more hydration products to fill the pores [24]. Therefore, when the SCS replacement levels were 10% and 20%, the mechanical strength development of the lightweight FGPs was better, and the flexural strength was also observed to follow the same trend. When the amount of foaming agent was increased to 2.0% and the curing time was 56 days, the flexural strength of the lightweight FGPs with SCS replacement levels of 10% and 20% was 0.40 and 0.50 MPa, respectively; in addition, Bai et al. (2018) used an H_2_O_2_ solution as the foaming agent and vegetable oil as the stabilizer to synthesize a foaming geopolymer. The results showed that when the H_2_O_2_ solution was 5 wt.% and the vegetable oil content was 20 wt.%, the bulk density was 0.37 g/cm^3^, the flexural strength was only 0.3 MPa [26], and the flexural strength was lower than that in our study of lightweight FGPs.

### 3.3. Thermal Conductivity Analysis of Lightweight Fgps

Table 4 shows the thermal conductivity of lightweight FGPs prepared with different SCS replacement levels, H_2_O_2_ solution addition levels, and curing times of 1–56 days. It can be seen from the table that when the curing time was 1 day, and the SCS replacement level was 0%, the thermal conductivity of the lightweight FGPs with amounts of added foaming agent of 0.5%, 1.0%, 1.5%, and 2.0% was 0.418, 0.314, 0.281, and 0.280 W/m × K, respectively. The results showed that the thermal conductivity of the lightweight FGPs decreased with increasing amounts of foaming agents. At a curing time of 28 days and an SCS replacement level of 0%, the thermal conductivity of the lightweight FGPs with amounts of added foaming agent of 0.5%, 1.0%, 1.5%, and 2.0% was 0.420, 0.297, 0.281, and 0.268 W/m × K, respectively, and this research results were consistent with those of Bergamonti et al. (2018) [27]. At a curing time of 1 day and an amount of added foaming agent of 0.5%, the thermal conductivity of the lightweight FGPs with SCS replacement levels of 10% and 20% was 0.451 and 0.570 W/m × K, respectively. The results show the thermal conductivity significantly dropped, which was due to the air bubbles that were generated inside the paste and that existed in the structure; the thermal conductivity of air is 0.173 W/m⋅K, and the thermal conductivity decreased with increasing amounts of air in the pores [25,27].

When the amount of foaming agent increased to 2.0%, the thermal conductivity of the lightweight FGPs with SCS replacement levels of 10% and 20% was 0.370 and 0.456 W/m⋅K, respectively. The results show that the redox reaction was very strong with increasing amounts of hydrogen peroxide in the system, causing more bubbles to be generated [22,23]. According to Du et al. (2016), a geopolymer with 10 wt.% SiC had a thermal conductivity of 0.9474 W/m⋅K [28], and the results of our study were better than those of Du et al. (2016). The lightweight FGPs obtained by adding SCS, proposed in this work, represent an innovative solution, which enhances the thermal resistance of the buildings, and also contributes to lower insulation costs. Moreover, due to environmental and energy concerns, the reuse of polyurethane foams from industrial wastes offers a sustainable waste recycling process, alternative to landfill or incineration.

### 3.4. Fire Resistance Properties of Lightweight Fgps

Table 5 shows the reverse-side temperature of the lightweight FGP prepared with different SCS replacement levels, added H_2_O_2_ solution levels, and curing times of 1–56 days. When the SCS replacement level was 0%, and the FGP was cured for 1 day, the reverse-side temperature of the lightweight FGPs with 0.5% and 1.0% added foaming agent were 322 and 294 °C, respectively, which showed that the reverse-side temperature of the lightweight FGPs drastically decreased with increasing amounts of foaming agent. After curing for 56 days, the reverse-side temperatures of lightweight FGPs with 1.0% and 2.0% added foaming agent were 244 and 273 °C, respectively. The results show that the reverse-side temperature gradually decreased with increasing curing time. Because the geopolymerization reaction continued to form more hydration products to increase the structural strength [24], the fire resistance performance of lightweight FGP materials has been improved.

When the curing time was 1 day, and the amount of added foaming agent was 0.5%, the reverse-side temperatures of the lightweight FGPs with SCS replacement levels of 10% and 20% were 286 and 311 °C, respectively. The results show that the reverse-side temperature significantly decreased because when the foaming agent was added, the air bubbles remaining in the paste created large pores, and the pores could effectively resist heat transfer [25,27]. When the amount of added foaming agent increased to 2.0%, the reverse-side temperatures of the lightweight FGPs with SCS replacement levels of 10% and 20% were 311 and 281 °C, respectively. The results show that the reverse-side temperature increased with increasing amounts of hydrogen peroxide due to the influence of the presence of SiC, while the chemical reaction will preferentially involve •OH to form CO_2_ [22,23]; in addition, as the k value of the O_2_ reaction decreased, the bubble content existing in the structure decreased. Wattanasiriwech et al. (2017) used mullite to prepare fireproof fly ash-based geopolymers. The results showed that after a 30 min fire resistance test, the reverse-side temperatures of geopolymers with mullite replacement levels of 40% and 60 wt.% were 473 and 435 °C, respectively. Therefore, although the presence of SiC sludge will reduce the fire resistance performance, the results of our study were better than those of Wattanasiriwech et al. (2017) [29].

### 3.5. FTIR Spectrum of Lightweight FGPs

**Figure 3:**
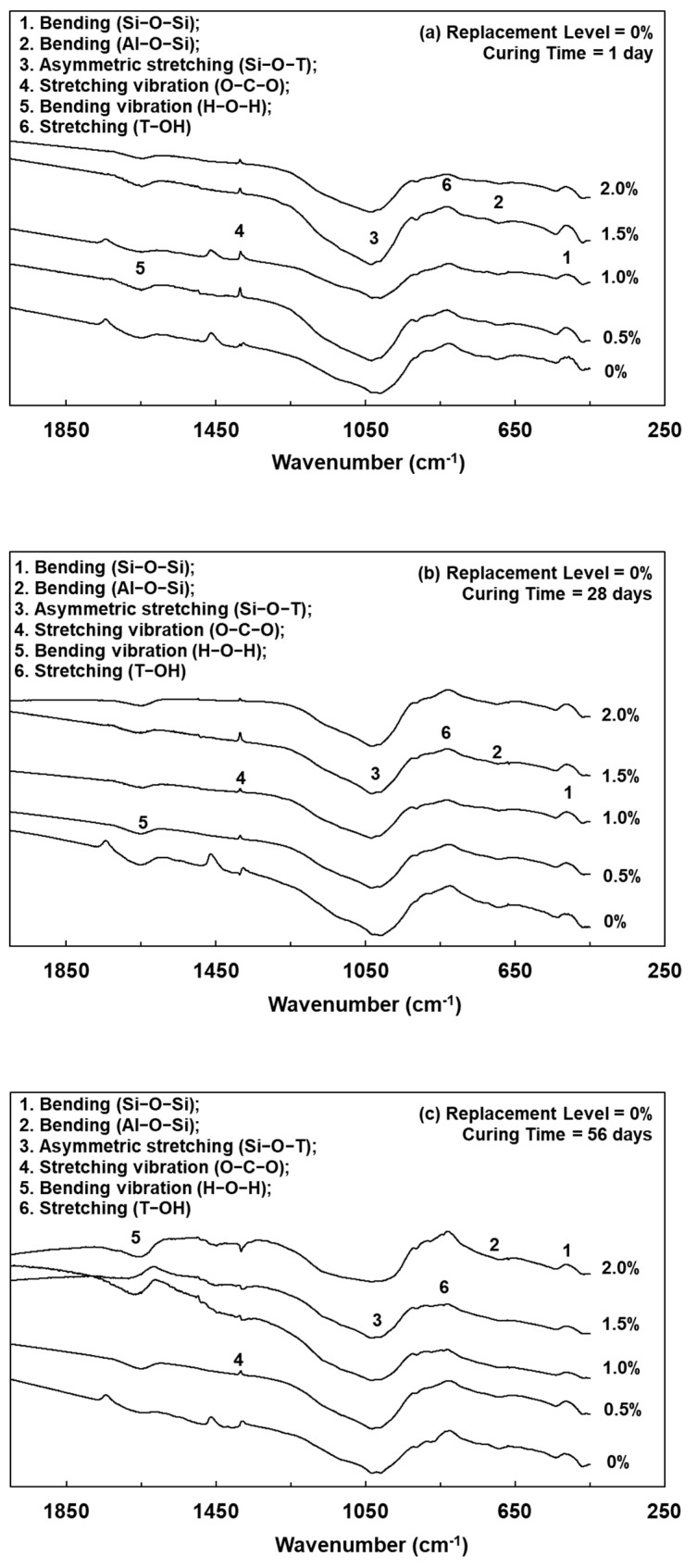
FTIR spectrums of lightweight FGPs. (Replacement level = 0%). (**a**) 1day; (**b**) 28 days and (**c**) 56 days.

**Figure 4:**
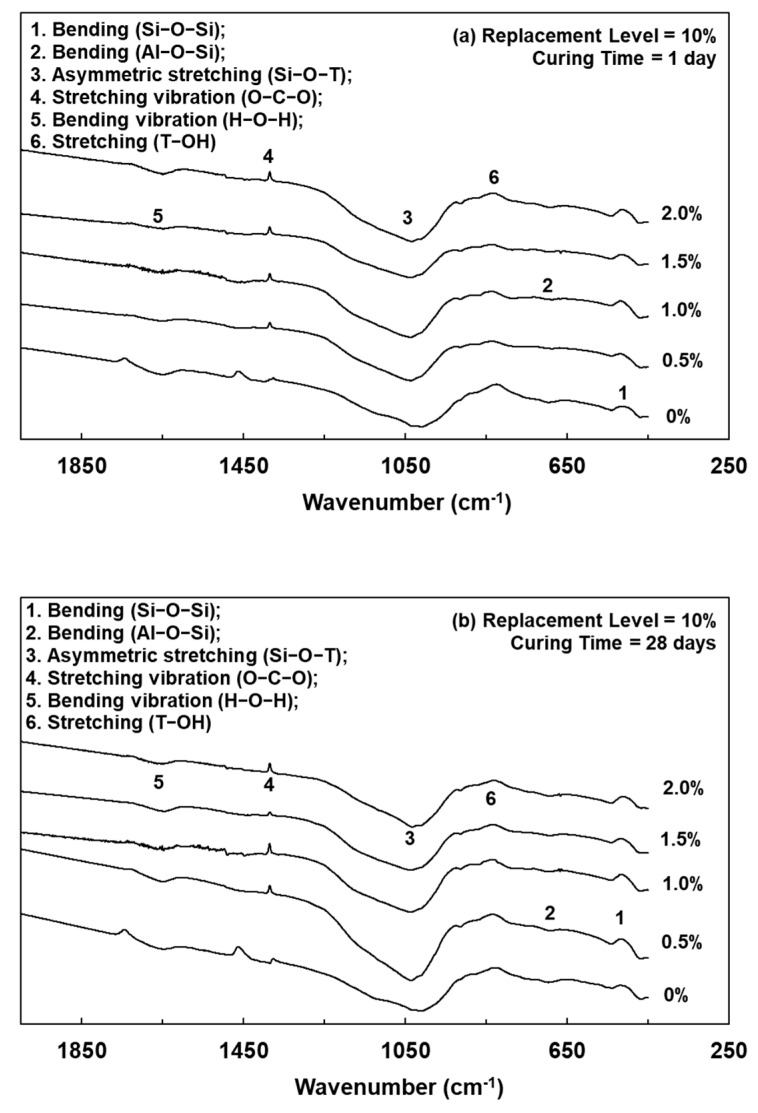

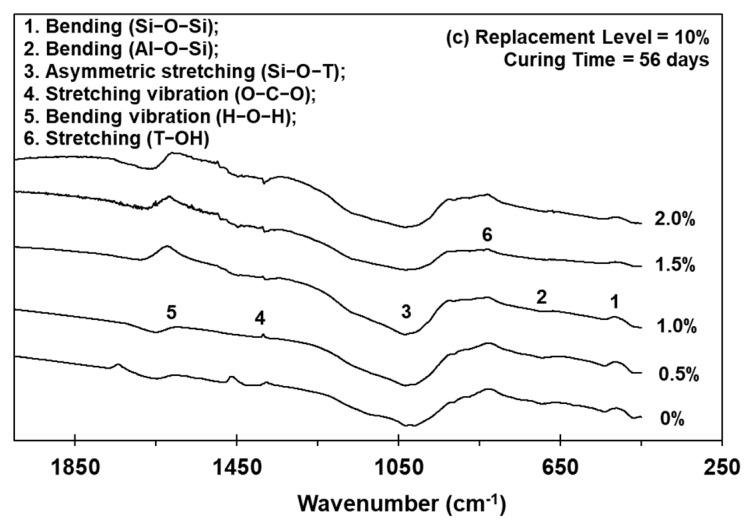
FTIR spectrums of lightweight FGPs. (Replacement level = 10%). (**a**) 1day; (**b**) 28 days and (**c**) 56 days.

**Figure 5:**
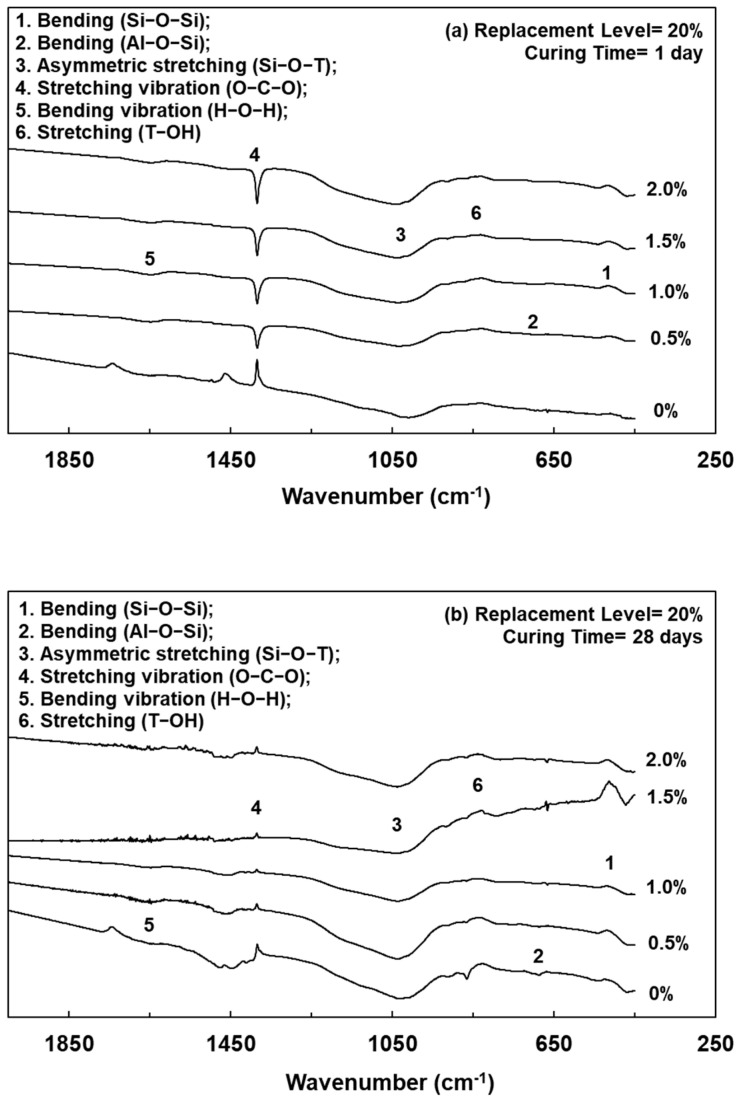

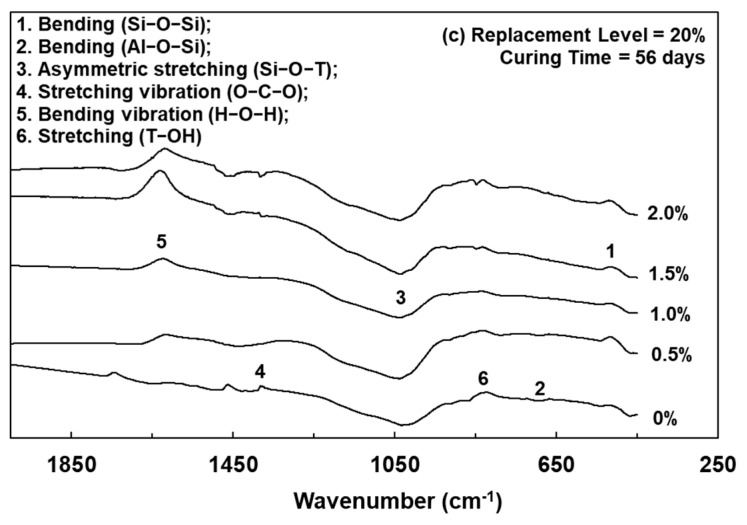
FTIR spectrums of lightweight FGPs. (Replacement level = 20%). (**a**) 1day; (**b**) 28 days and (**c**) 56 days.

Lightweight FGPs with different SCS replacement levels and added H_2_O_2_ solution levels were analyzed by FTIR spectrum, as shown in Figure 3, Figure 4 and Figure 5. It can be seen from Figure 5 that when the SCS replacement level was 0% and after curing for 1 day, the sample without any added foaming agent had bands at 1028, 700, and 470 cm^−1^, corresponding to Si–O–T asymmetric bonding (where T = Al or Si), Al–O–Si bonding and Si–O–Si bonding, respectively [20]. When the amount of added foaming agent was 0.5%, 1.0%, 1.5%, and 2.0%, the same bands were observed. When the curing age was 56 days, after geopolymerization of the lightweight FGP material, the wavenumber of the peak corresponding to Si–O–T asymmetric bonding at 1028 cm^−1^ shifted to 998 cm^−1^. The results show that the peak attributed to Si–O–T asymmetric bonding shifted to a lower wavenumber with increasing curing time.

Figure 4 and Figure 5 show that when a lightweight FGP with an amount of added foaming agent of 1.0% was cured for 1 day and the SCS replacement levels increased from 10% to 20%, the peaks corresponding to Si–O–T were located at 1032 and 1039 cm^−1^, respectively. This shows that the bands had not significantly changed with the addition of hydrogen peroxide because the amount of added H_2_O_2_ solution did not change the rate of geopolymerization [18]. However, the intensity of the band at approximately 1416 cm^−1^ significantly increased with increasing amounts of foaming agent, and this peak corresponded to an O–C–O asymmetric stretching vibration in the ${\mathrm{CO}}_{3}^{2-}$ group [2]. When SiC was present in this environment, the CO_2_ concentration in the system increased, and carbonation of the unreacted alkaline substances occurred, thereby enhancing the band strength of the O–C–O asymmetric stretching vibration. Therefore, when the SCS replacement level was 20% and the amount of added foaming agent was 2.0%, the intensity of the band at approximately 1416 cm^−1^ significantly increased.

### 3.6. SEM Observation of Lightweight FGPs

**Figure 6:**
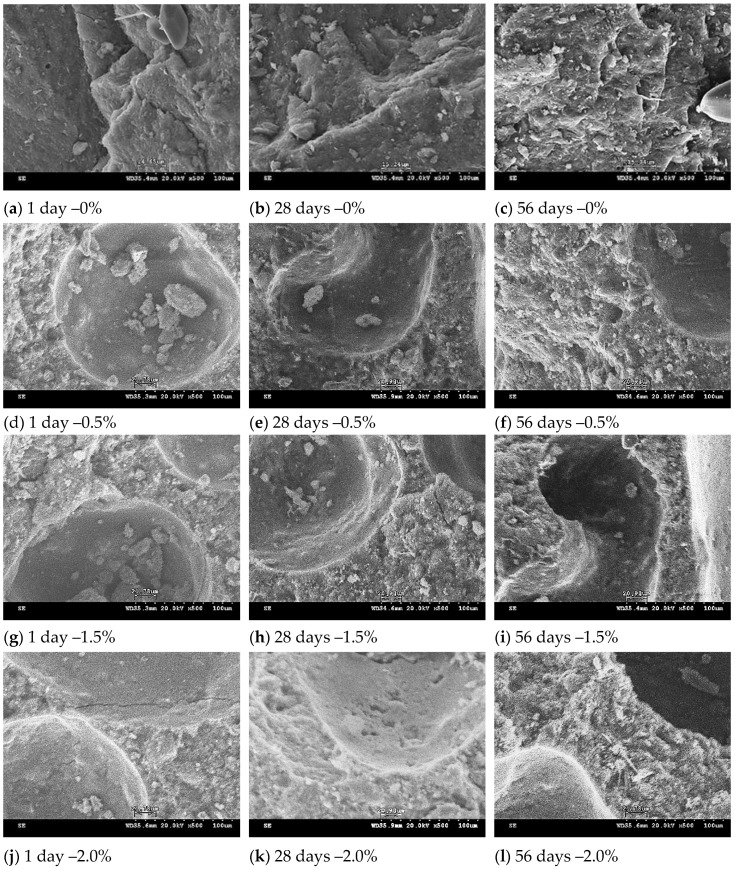
SEM micrograph of lightweight FGPs. (Replacement level = 0%). H_2_O_2_ solution levels: (**a**–**c**) 0%; (**d**–**f**) 0.5%; (**g**–**i**) 1.5% and (**j**–**l**) 0.5%.

**Figure 7:**
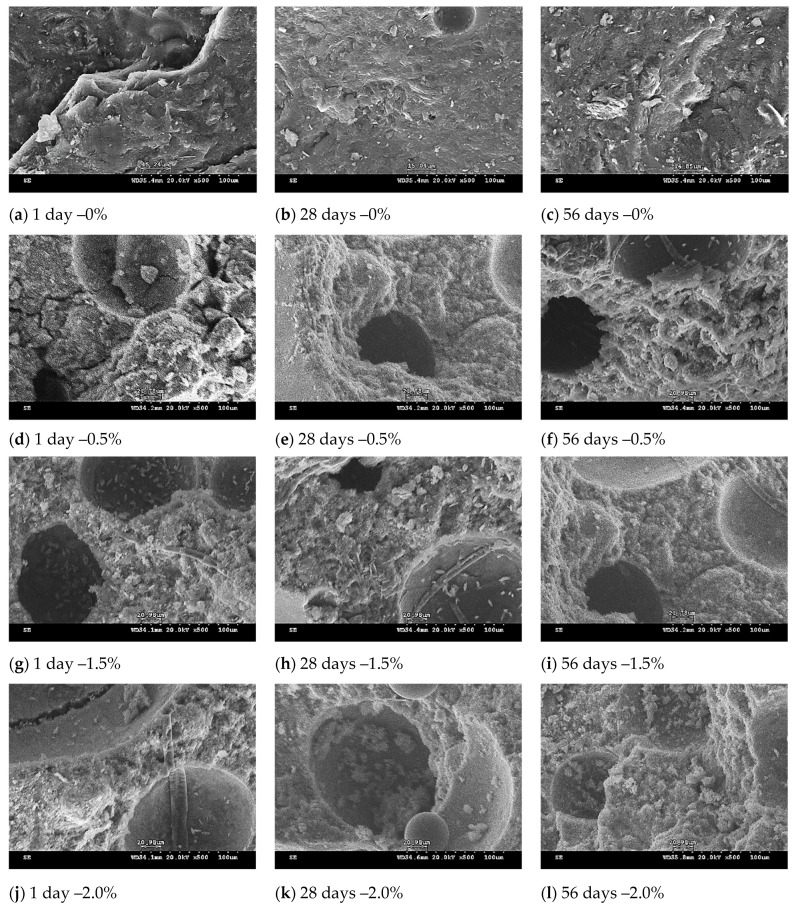
SEM micrograph of lightweight FGPs. (Replacement level = 10%). H_2_O_2_ solution levels: (**a**–**c**) 0%; (**d**–**f**) 0.5%; (**g**–**i**) 1.5% and (**j**–**l**) 0.5%.

**Figure 8:**
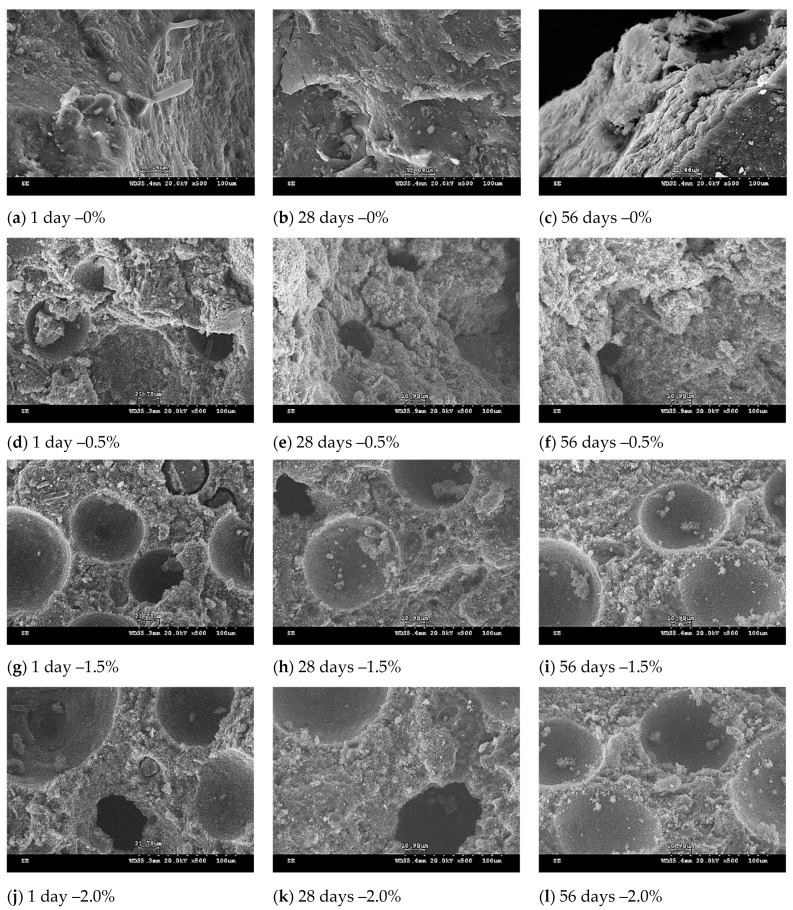
SEM micrograph of lightweight FGPs. (Replacement level = 20%). H_2_O_2_ solution levels: (**a**–**c**) 0%; (**d**–**f**) 0.5%; (**g**–**i**) 1.5% and (**j**–**l**) 0.5%.

Figure 6, Figure 7 and Figure 8 showed the microstructure of the lightweight FGPs with different SCS replacement levels, different added H_2_O_2_ solution levels, and curing times of 1–56 days. The results show that for lightweight FGPs with an SCS replacement level of 0%, an amount of added foaming agent of 0.5%, and a curing time of 1 day, the bubbles remaining in the lightweight FGP material creates voids (large pores), which was observed after adding the foaming agent, but the edge angle structure was still be observed. When the amount of added foaming agent was 2.0%, and the SCS replacement level was 10%, the sample showed dispersed large pores, which was attributed to the bubbles remaining in the paste during dissolution and the polycondensation reaction [27]. In addition, plate-like particles were also observed in the structure because the amount of added H_2_O_2_ solution did not change the rate of geopolymerization [18].

When the SCS replacement level was 20%, many small pores were regularly distributed in the matrix. According to Petlitckaia et al. (2019), the decomposition of H_2_O_2_ may be affected by the chemical composition of the geopolymer [19]. As the amount of added H_2_O_2_ solution increased to 2%, it could be observed that the number of pores gradually increased, and the porosity ratio of the lightweight FGP with an irregular structure increased. The results show that the redox reaction was very strong with increasing amounts of hydrogen peroxide in the system, causing more bubbles to be generated [22,23], but the hole sizes gradually changed from large holes to small holes in the internal structure. Characteristic fireproof materials have a high porosity ratio; the porosity ratio is important to obtain fire resistance, which depends on the amount of SiC sludge added to the lightweight FGP materials. In addition, none of the samples were observed to contain microcracks in their structures. The samples had a suitable uniformity and interconnected pore distribution during the curing time of 56 days, and the structure appeared to be moderately dense.

### 3.7. Foaming Kinetic Analysis of The Lightweight FGP

Due to their effect on mechanical strength, the number and structure of pores are important parameters of porous materials [18,26]. Therefore, this study first explored the effects of different SCS, MK, and H_2_O_2_ foaming agent contents on the foaming kinetic of lightweight FGPs. Novais et al. (2016) indicated that the rate of geopolymerization depends on the concentration of the alkali activator solution and the liquid/solid (L/S) ratio, and the amount of added H_2_O_2_ solution does not change the rate of geopolymerization. However, the volume expansion, bulk density, homogeneity, and the final properties of the lightweight FGP varied significantly with varying H_2_O_2_ contents [18]. First, H_2_O_2_ was thermodynamically unstable in the basic medium, and it easily decomposed into water and oxygen (O_2_) [19], as shown in Equation (1). When SiC was present in this environment, the H_2_O_2_ reaction was more likely to release •OH, as shown in Equation (2). Si atoms could penetrate into the paste of the FGP, and the generated hydroxyl radical (•OH) is a strong oxide species; additionally, the chemical reaction will preferentially react with •OH to generate silicon dioxide (SiO_2_), H_2_O, and carbon dioxide (CO_2_) [22,23], as shown in Equation (3). The bubbles remaining in the paste will expand and create voids (large pores). Therefore, the volumetric expansion of the lightweight FGP prepared in this research, which depends on the amount of gas produced by the following reaction, was examined:

(1) $$H_{2}O_{2}\to H_{2}O+\frac{1}{2}O_{2}$$

(2) $$H_{2}O_{2}\to 2{\mathrm{OH}}^{˙}$$

(3) $$\mathrm{SiC}+4{\mathrm{OH}}^{˙}+O_{2}\to {\mathrm{SiO}}_{2}+2H_{2}O+{\mathrm{CO}}_{2}$$

**Figure 9:**
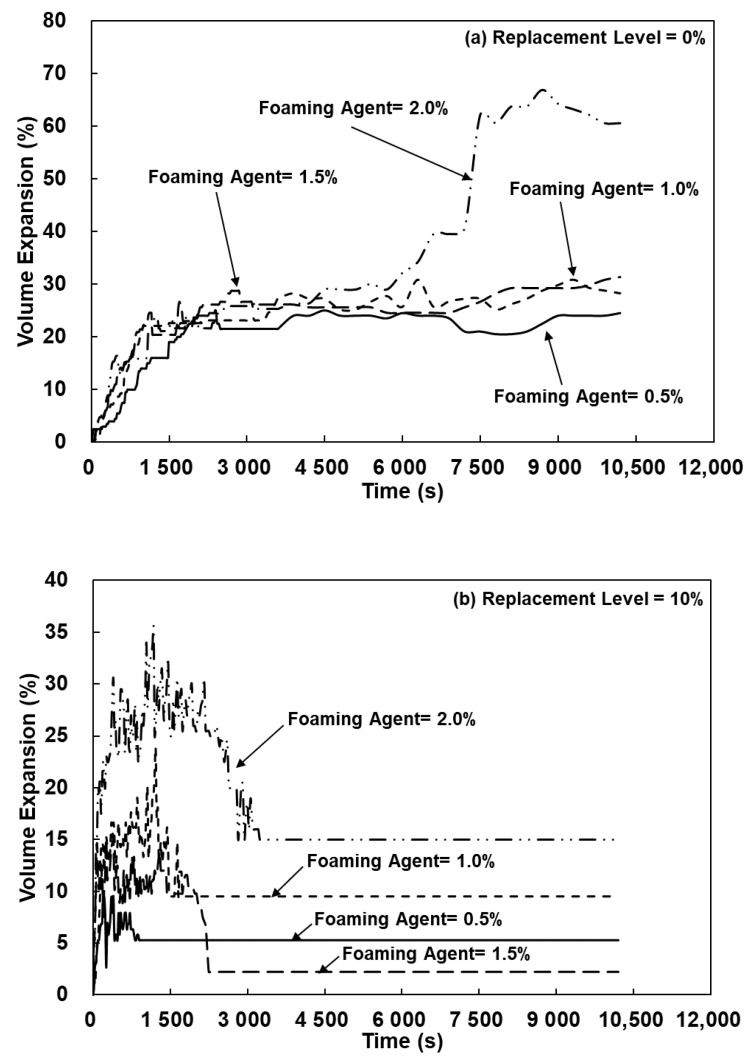

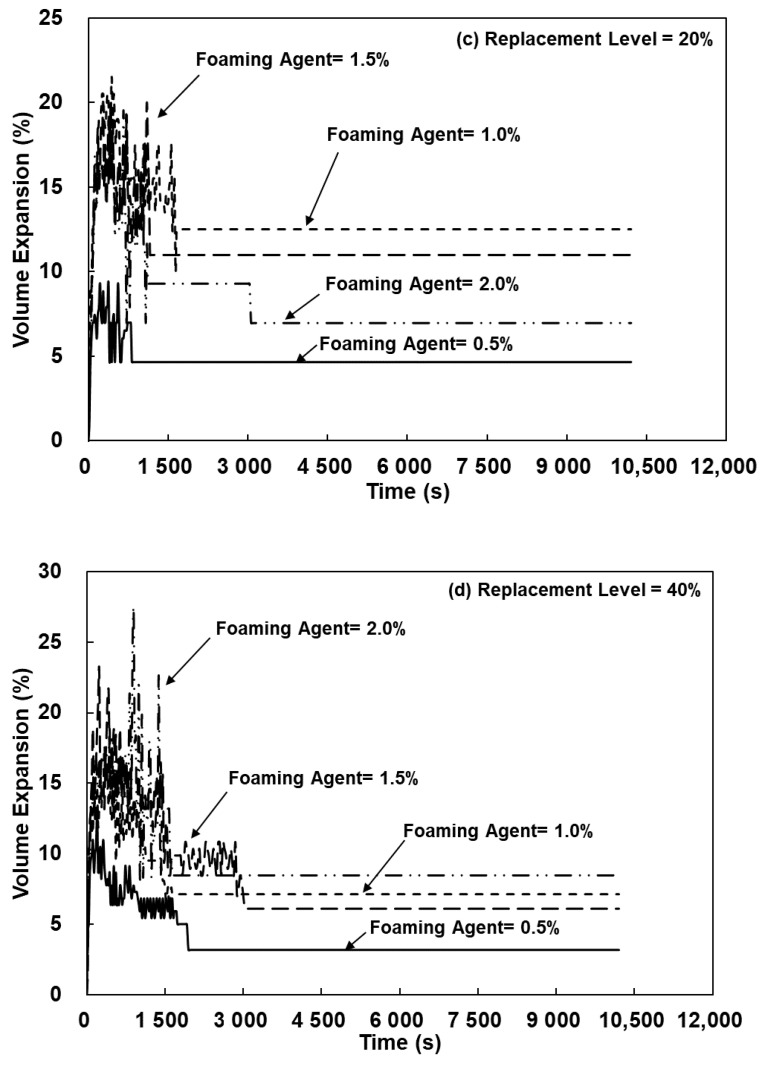
The volume expansion ratio of lightweight FGPs with SCS replacement levels. (**a**) 0%; (**b**) 10%; (**c**) 20% and (**d**) 40%.

Figure 9 shows the volume expansion ratio of the lightweight FGP prepared with different SCS replacement levels and added H_2_O_2_ solution levels. It was known from Figure 9a that when the SCS replacement level was 0%, and the addition amount of the H_2_O_2_ solution was 0.5%, the volume expansion rate increased with increased reaction times. Because H_2_O_2_ was thermodynamically unstable in the basic medium, it easily decomposed into water and oxygen [19]. As the amount of added H_2_O_2_ solution increased from 1.0% to 2.0%, the volume expansion ratios of the lightweight FGPs increased from 8.21% to 60.53% (Figure 9a). This showed that the higher the initial concentration of the H_2_O_2_ solution is, the greater the volume expansion ratio, and our research results were consistent with previous research [18]. In addition, when the SCS replacement levels and the added H_2_O_2_ solution amount were 10% and 0.5%, the volume expansion ratio of the lightweight FGP was 5.26%. As the added amount of H_2_O_2_ solution was increased to 2%, the volume expansion ratio of the lightweight FGP increased to 15.00% because H_2_O_2_ was continuously reacted to form water and oxygen in the system. When the added amount of H_2_O_2_ solution was 0.5%, and the SCS replacement levels were 20–40%, the volume expansion ratio of the lightweight FGPs gradually decreased to 4.65%, 4.76%, and 3.20%. When the silicon carbide sludge existed in this environment, the H_2_O_2_ reaction tended to release •OH, and its redox reaction was very strong, accelerating the decomposition of H_2_O_2_ and O_2_ [21,22]; therefore, it was difficult to control the morphology, size, particle-size distribution and porosity of the FGP, resulting in a lightweight FGP with a weak and unstable structure after foaming. Hence, when the SCS replacement levels were 20–40%, and the added amount of H_2_O_2_ solution was 2.0%, the volume expansion ratios of the lightweight FGPs were 6.98–8.49%, which were all below the 0% and 10% SCS replacement levels of the lightweight FGPs, as shown in Figure 9.

The results demonstrated the different foaming kinetics of lightweight FGPs, for which were two possible explanations. First, a synergistic effect existed between silicon carbide sludge and metakaolin, the geopolymerization reaction of metakaolin was dominant, and the addition of silicon carbide sludge provided more reaction paths [24]. Second, according to the research of Petlitckaia et al. (2019), the decomposition of H_2_O_2_ may be affected by the chemical composition of the geopolymer [19]. In fact, the pH value or the presence of transition metals or metal oxides has a great influence on the kinetics of oxygen production [19]. Suppose that all samples have the same amount of H_2_O_2_ added; any difference must be attributed to the different SiC sludge replacement levels. According to Equation (1) to Equation (3), when the SCS joined the chemical reaction system, 2 mols of H_2_O_2_ and 1 mol of O_2_ were consumed to form 1 mol of CO_2_, and then, the decomposition of H_2_O_2_ and O_2_ was accelerated. Based on the abovementioned experimental results and the stoichiometry of the decomposition reaction of H_2_O_2_, the amount of O_2_ produced over time was calculated. The H_2_O_2_ concentration can be determined from the first-order reaction [19], which was obtained from Equations (4) and (5), where k was the first-order reaction rate constant (s^−1^), and [H_2_O_2_]_0_ and [H_2_O_2_] were the initial H_2_O_2_ concentrations at time t_0_ and t, respectively.

(4) $$v=-\frac{d\left[H_{2}O_{2}\right]}{\mathrm{dt}}=k\cdot \left[H_{2}O_{2}\right]$$

(5) $$\left[H_{2}O_{2}\right]={\left[H_{2}O_{2}\right]}_{0}\cdot e^{-\mathrm{kt}}$$

Subsequently, the following equation can be used to calculate the generated oxygen concentration [O_2_] for the experimental data comparison:

(6) $$\left[O_{2}\right]=\frac{{\left[H_{2}O_{2}\right]}_{0}}{2}\left(1-e^{-\mathrm{kt}}\right)$$

**Figure 10:**
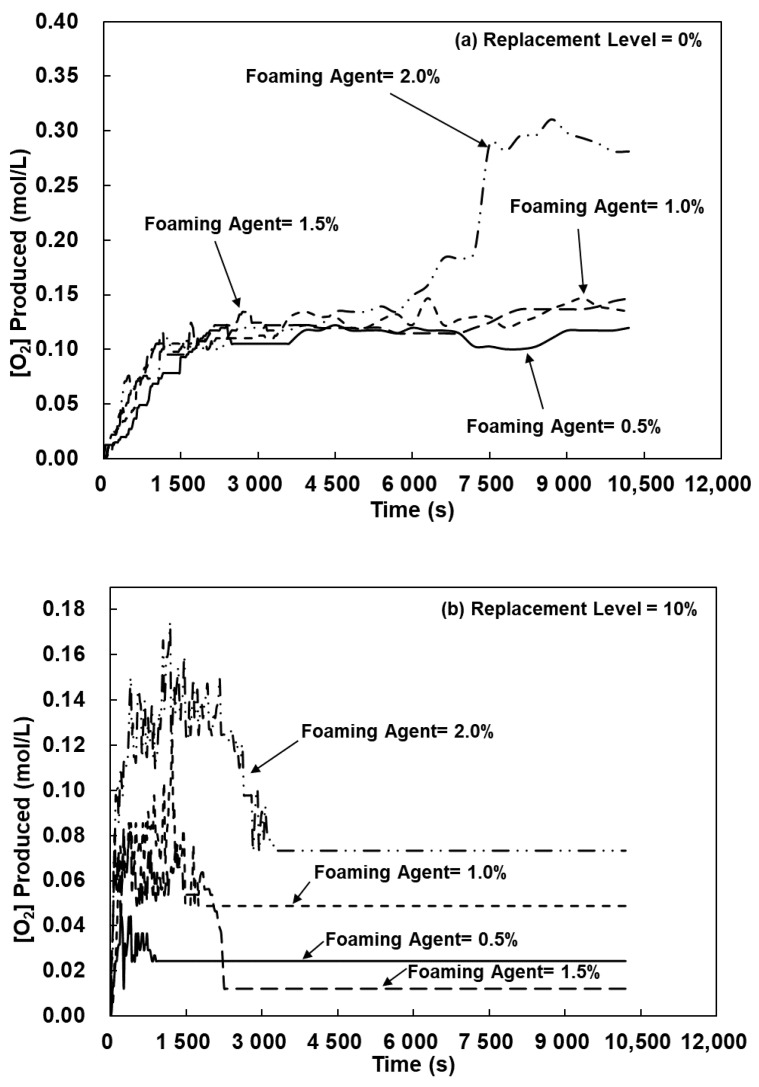

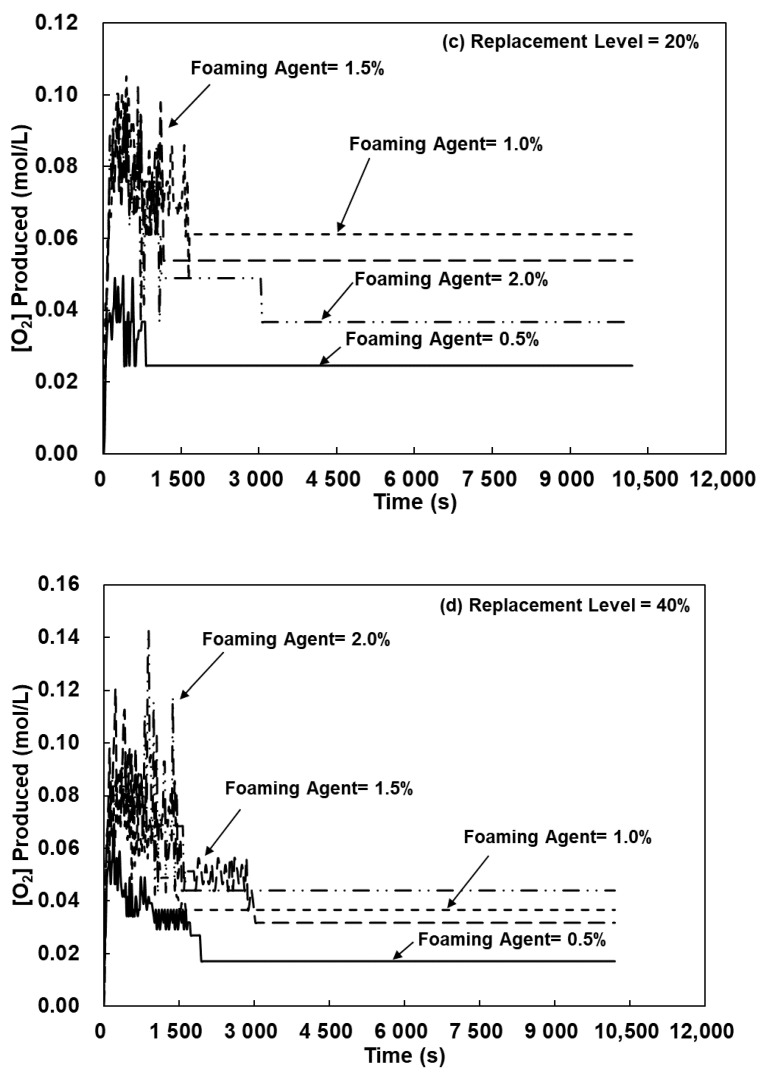
The amount of O_2_ produced for the lightweight FGPs with SCS replacement levels. (**a**) 0%; (**b**) 10%; (**c**) 20% and (**d**) 40%.

According to Equations (5) and (6), the k value of the first-order reaction rate constant and oxygen concentration [O_2_] value were calculated, and the results are listed in Table 6 and Figure 10. The results showed that when the SCS replacement level was 0% and the added H_2_O_2_ solution amount was 0.5%, the oxygen concentration [O_2_] was 0.12 mol/L; the oxygen concentration [O_2_] increased to 0.281 mol/L when the amount of added hydrogen peroxide was 2.0%, and the k value of the first-order reaction increased from 3.82 × 10^−4^ to 4.69 × 10^−4^. This showed that increasing the concentration of H_2_O_2_ in the system promoted the decomposition of H_2_O_2_ into water and O_2_, and our research results were consistent with those of Petlitckaia et al. (2019) [19]. Additionally, the results of the foaming kinetic analysis of the lightweight FGP with 10% SCS replacement levels showed that when the added H_2_O_2_ solution amount increased from 0.5% to 2.0%, the O_2_ concentration increased from 0.024 to 0.073 mol/L. However, due to the influence of the presence of SiC, the chemical reaction will preferentially involve •OH to form CO_2_ [22,23], and the k value of the O_2_ reaction decreased from 2.94 × 10^−4^ to 1.76 × 10^−4^. When the amount of added H_2_O_2_ solution was 2.0%, and the SCS replacement levels were 20–40%, the O_2_ concentrations were 0.037, 0.049, and 0.044 mol/L, which showed that adding too much SCS caused the reaction kinetics to preferentially form CO_2_. Therefore, when the SCS replacement levels were 20–40%, the reaction consumed O_2_ to form CO_2_ in the system, while the k value of the O_2_ reaction was lower than that of the samples with 0% and 10% SCS replacement levels, in which the rate constants were 1.76 × 10^−4^, 1.36 × 10^−4^, and 1.76 × 10^−4^ (Table 6).

For the abovementioned reasons, the volume expansion ratio gradually increased with the addition of hydrogen peroxide, and the reaction system was affected by the presence of SiC sludge, which caused the reaction to consume O_2_ to form CO_2_. Increasing the SCS replacement levels of the lightweight FGP will decrease the k value of the reaction and oxygen concentration. Therefore, this study considered the application of silicon carbide sludge as a follow-up study of lightweight FGPs and will use SCS replacement levels of 0–20% for the experimental analysis.

## 4. Conclusions

This study used an H_2_O_2_ solution and SiC sludge to prepare lightweight foaming geopolymer materials by the direct foaming method. The contents of H_2_O_2_ solution and SiC sludge were evaluated to determine their influence on foaming kinetic. Results showed that when the SCS replacement level was 10%, and the amount of added foaming agent was 2.0%, the porosity ratio of the lightweight FGP increased from 31.88% to 40.03%. The mechanical strength of the lightweight FGPs with SCS replacement levels of 10% and 20% was 0.88 and 0.31 MPa, respectively. When the amount of foaming agent increased to 2.0%, the thermal conductivity of the lightweight FGPs with SCS replacement levels of 10% and 20% were 0.370 and 0.456 W/m⋅K, respectively. Additionally, when the curing time was 1 day, and the amount of added foaming agent was 0.5%, the reverse-side temperature of the lightweight FGPs with SCS replacement levels of 10% and 20% were 286 and 311 °C, respectively. The k value of the O_2_ reaction decreased from 2.94 × 10^−4^ to 1.76 × 10^−4^. This is due to the reaction system being affected by the presence of SiC sludge, which was caused the reaction to consume O_2_ to form CO_2_. The results have been proposed to explain that the successfully manufactured lightweight FGPs had a low thermal conductivity (0.370–0.456 W/m⋅K). This study presented the potential for lightweight FGPs geopolymer applications for the construction industry.

## Figures and Tables

**Table 1:** The composition of materials.

Composition	SCS	Kaolinite	Metakaolin
SiO_2_ (%)	75.40	53.70	51.80
Al_2_O_3_ (%)	0.80	37.88	43.00
Fe_2_O_3_ (%)	0.58	0.88	1.30
CaO (%)	0.09	0.20	0.25
SO_3_ (%)	0.06	-	-
Na_2_O (%)	-	0.04	0.04
K_2_O (%)	0.01	0.34	0.32
SiC (%)	23.00	-	-

**Table 2:** Compressive strength development of lightweight FGPs.

SCS (wt. %)	Foaming Agent (vol. %)	Compressive Strength (MPa)				
		1 Day	7 Days	14 Days	28 Days	56 Days
0	0.0	38.28	40.49	41.31	43.08	44.02
	0.5	1.32	2.03	2.33	2.46	2.50
	1.0	0.27	0.59	0.60	0.61	0.80
	1.5	0.23	0.33	0.52	0.56	0.63
	2.0	0.08	0.06	0.16	0.29	0.29
10	0.0	33.77	34.12	34.80	36.26	35.05
	0.5	1.25	2.09	2.19	2.81	3.15
	1.0	0.23	0.38	0.60	0.79	1.29
	1.5	0.08	0.29	0.54	0.69	1.25
	2.0	0.05	0.18	0.47	0.54	0.88
20	0.0	25.32	27.06	27.38	28.43	29.94
	0.5	0.78	1.18	1.50	1.58	2.42
	1.0	0.69	1.03	1.22	1.39	2.01
	1.5	0.07	0.26	0.27	0.33	0.60
	2.0	0.05	0.06	0.08	0.18	0.31

**Table 3:** Flexural strength development of lightweight FGPs.

SCS (wt. %)	Foaming Agent (vol. %)	Flexural Strength (MPa)				
		1 Day	7 Days	14 Days	28 Days	56 Days
0	0.0	4.19	4.55	4.73	4.74	5.18
	0.5	0.80	1.25	1.35	1.35	1.35
	1.0	0.50	0.65	0.70	0.75	0.85
	1.5	0.30	0.30	0.50	0.50	0.70
	2.0	0.25	0.30	0.35	0.40	0.45
10	0.0	4.05	4.55	4.70	4.83	5.22
	0.5	0.65	0.80	1.10	1.20	1.20
	1.0	0.30	0.40	0.40	0.45	0.70
	1.5	0.15	0.25	0.34	0.40	0.50
	2.0	0.10	0.25	0.25	0.35	0.40
20	0.0	3.97	3.98	4.58	4.63	4.74
	0.5	0.80	0.90	1.00	1.10	1.30
	1.0	0.60	0.80	1.00	1.05	1.05
	1.5	0.40	0.40	0.50	0.60	0.75
	2.0	0.20	0.30	0.40	0.44	0.50

**Table 4:** Thermal conductivity of lightweight FGPs.

SCS (wt. %)	Foaming Agent (vol. %)	Thermal Conductivity (W/m × K)				
		1 Day	7 Days	14 Days	28 Days	56 Days
0	0.0	0.757	0.742	0.794	0.739	0.720
	0.5	0.418	0.379	0.436	0.420	0.366
	1.0	0.314	0.302	0.322	0.297	0.257
	1.5	0.281	0.280	0.313	0.281	0.255
	2.0	0.280	0.280	0.270	0.268	0.207
10	0.0	0.796	0.751	0.776	0.730	0.686
	0.5	0.451	0.465	0.450	0.439	0.368
	1.0	0.447	0.431	0.404	0.376	0.365
	1.5	0.392	0.385	0.375	0.337	0.333
	2.0	0.389	0.382	0.370	0.334	0.323
20	0.0	0.799	0.764	0.752	0.721	0.695
	0.5	0.570	0.563	0.491	0.449	0.445
	1.0	0.564	0.531	0.469	0.447	0.423
	1.5	0.561	0.491	0.462	0.434	0.413
	2.0	0.557	0.491	0.456	0.434	0.405

**Table 5:** Reverse-side temperature of lightweight FGPs.

SCS (wt. %)	Foaming Agent (vol. %)	Reverse-Side Temperature (°C)		
		1 Day	28 Days	56 Days
0	0.0	415	353	357
	0.5	322	252	306
	1.0	294	234	244
	1.5	308	251	268
	2.0	346	317	273
10	0.0	347	367	306
	0.5	286	285	241
	1.0	318	301	269
	1.5	332	360	298
	2.0	340	373	311
20	0.0	358	321	310
	0.5	311	250	249
	1.0	344	310	255
	1.5	367	333	278
	2.0	383	340	281

**Table 6:** The chemical reaction parameters and reaction rates of lightweight FGPs.

SCS (wt. %)	Foaming Agent (vol. %)	V_0_ (mL)	V_H2O2_ (mL)	VE (%)	Volume Fraction of Gas (ϕ_g_)	k (s^−1^)
0	0.5	20.0	4.9	24.50	0.197	3.82 × 10^−4^
	1.0	19.5	5.5	28.21	0.220	4.62 × 10^−4^
	1.5	19.1	6.0	31.41	0.239	4.72 × 10^−4^
	2.0	19.0	11.5	60.53	0.377	4.69 × 10^−4^
10	0.5	19.0	1.0	5.26	0.050	2.94 × 10^−4^
	1.0	21.0	2.0	9.52	0.087	2.04 × 10^−4^
	1.5	22.5	0.5	2.22	0.022	1.88 × 10^−4^
	2.0	20.0	3.0	15.00	0.130	1.76 × 10^−4^
20	0.5	21.5	1.0	4.65	0.044	2.26 × 10^−4^
	1.0	20.0	2.5	12.50	0.111	1.58 × 10^−4^
	1.5	20.0	2.2	11.00	0.099	1.46 × 10^−4^
	2.0	21.5	1.5	6.98	0.065	1.76 × 10^−4^
30	0.5	21.0	1.0	4.76	0.045	1.36 × 10^−4^
	1.0	21.2	1.3	6.13	0.058	1.16 × 10^−4^
	1.5	19.0	2.0	10.53	0.095	1.36 × 10^−4^
	2.0	21.0	2.0	9.52	0.087	1.36 × 10^−4^
40	0.5	21.9	0.7	3.20	0.031	3.31 × 10^−4^
	1.0	21.0	1.5	7.14	0.067	1.18 × 10^−4^
	1.5	21.2	1.3	6.13	0.058	2.12 × 10^−4^
	2.0	21.2	1.8	8.49	0.078	1.76 × 10^−4^

## Data Availability

The data presented in this study are available on request from the corresponding author.

## References

[1] Yoko A., Oshima Y.S. (2013). Recovery of silicon from silicon sludge using supercritical water. J. Supercrit. Fluids.

[2] Lo K.W., Lin K.L., Cheng T.W., Zhang B.X. (2019). The influence of sapphire substrate silicon carbide sludge on structural properties of metakaolin-based geopolymers. Environ. Prog. Sustain. Energy.

[3] Tsai T.H. (2011). Silicon sawing waste treatment by electrophoresis and gravitational settling. J. Hazard. Mater..

[4] Lan A., Liu C.E., Yan H.L., Yua H.T., Li I.T., Hsua H.P., Lan C.W. (2019). Silicon ingot casting using reusable silicon nitride crucibles made from diamond wire sawing kerf-loss silicon. J. Cryst. Growth.

[5] Davidovits J. (2011). Geopolymer Chemistry and Applications.

[6] Moni S.M.F.K., Ikeora O., Pritzel C., Görtz B., Trettin R. (2020). Preparation and properties of fly ash based geopolymer concrete with alkaline waste water obtained from foundry sand regeneration process. J. Mater. Cycles Waste Manag..

[7] Moutinho S., Costa C., Andrejkovičová S., Mariz L., Sequeira C., Terroso D., Rocha F., Velosa A. (2020). Assessment of properties of metakaolin-based geopolymers applied in the conservation of tile facades. Constr. Build. Mater..

[8] Zhang J., Li S.C., Li Z.F., Liu C., Gao Y.F. (2020). Feasibility study of red mud for geopolymer preparation: Effect of particle size fraction. J. Mater. Cycles Waste Manag..

[9] Shi J.Y., Liu B.J., Liu Y.C., Wang E.L., He Z.H., Xu H.J., Ren X.D. (2020). Preparation and characterization of lightweight aggregate foamed geopolymer concretes aerated using hydrogen peroxide. Constr. Build. Mater..

[10] Bhogayata A., Dave S.V., Arora N.K. (2020). Utilization of expanded clay aggregates in sustainable lightweight geopolymer concrete. J. Mater. Cycles Waste Manag..

[11] Petlitckaia S., Barré Y., Piallat T., Grauby O., Ferry D., Poulesquen A. (2020). Functionalized geopolymer foams for cesium removal from liquid nuclear waste. J. Clean. Prod..

[12] Irshidat M.R., Abdel-Jawad Y.A., Al-Sughayer R. (2018). Feasibility of producing sustainable geopolymer composites made of locally available natural pozzolan. J. Mater. Cycles Waste Manag..

[13] Murri A.N., Miccio F., Medri V., Landi E. (2020). Geopolymer-composites with thermomechanical stability as oxygen carriers for fluidized bed chemical looping combustion with oxygen uncoupling. Chem. Eng. J..

[14] Tan T.H., Mo K.H., Ling T.C., Lai S.H. (2020). Current development of geopolymer as alternative adsorbent for heavy metal removal. Environ. Technol. Innov..

[15] Xu M.X., He Y., Liu Z.H., Tong Z.F., Cui X.M. (2019). Preparation of geopolymer inorganic membrane and purification of pulp-papermaking green liquor. Appl. Clay Sci..

[16] Zhang Y.J., Han Z.C., He P.Y., Chen H. (2020). Geopolymer-based catalysts for cost-effective environmental governance: A review based on source control and end-of-pipe treatment. J. Clean. Prod..

[17] Kinnunen P., Yliniemi J., Talling B., Illikainen M. (2017). Rockwool waste in fly ash geopolymer composites. J. Mater. Cycles Waste Manag..

[18] Novais R.M., Buruberri L.H., Ascensão G., Seabra M.P., Labrincha J.A. (2016). Porous biomass fly ash-based geopolymers with tailored thermal conductivity. J. Clean. Prod..

[19] Petlitckaia S., Poulesquen A. (2019). Design of lightweight metakaolin based geopolymer foamed with hydrogen peroxide. Ceram. Int..

[20] Novais A.G., Rui M., Buruberri L.H., Senff L., Labrincha J.A. (2016). Influence of blowing agent on the fresh- and hardered-state properties of lightweight geopolymer. Mater. Des..

[21] Singh S.P., Namdeo H., Kumar B.S.M. (2020). Strength characteristics of lightweight geopolymer. Geotech. Charact. Model..

[22] Shi X.L., Pan G.S., Zhou Y., Gu Z.H., Gong H., Zou C.L. (2014). Characterization of colloidal silica abrasives with different sizes andtheir chemical-mechanical polishing performance on 4H-SiC (0 0 0 1). Appl. Surf. Sci..

[23] Kubota A., Yoshimura M., Fukuyama S., Iwamoto C., Touge M. (2012). Planarization of C-face 4H-SiC substrate using Fe particles and hydrogen peroxide solution. Precis. Eng..

[24] Hu Y., Liang S., Yang J.K., Chen Y., Ye N., Ke Y., Tao S.Y., Xiao K.K., Hu J.P., Hou H.J. (2012). Role of Fe species in geopolymer synthesized from alkali-thermal pretreated Fe-rich Bayer red mud. Constr. Build. Mater..

[25] Shiu H., Lin K., Chao S., Hwang C., Cheng T. (2014). Effects of foam agent on characteristics of thin-film transistor liquid crystal display waste glass-metakaolin-based cellular geopolymer. Environ. Prog. Sustain. Energy.

[26] Bai C.Y., Ni T., Wang Q.L., Li H.Q., Colombo P. (2018). Porosity, mechanical and insulating properties of geopolymer foams using vegetable oil as the stabilizing agent. J. Am. Ceram. Soc..

[27] Bergamonti L., Taurino R., Cattani L., Ferretti D., Bondioli F. (2018). Lightweight hybrid organic-inorganic geopolymers obtained using polyurethane waste. Constr. Build. Mater..

[28] Du F.P., Xie S.S., Zhang F., Tang C.Y., Chen L., Law W.C., Tsui C.P. (2016). Microstructure and compressive properties of silicon carbide reinforced geopolymer. Compos. Part B Eng..

[29] Wattanasiriwech S., Nurgesang F.A., Wattanasiriwech D., Timakul P. (2017). Characterisation and properties of geopolymer composite part 1: Role of mullite reinforcement. Ceram. Int..

